# Early Childhood Educators’ Knowledge, Self-Efficacy and Risk Tolerance for Outdoor Risky Play Following a Professional Risk Re-Framing Workshop

**DOI:** 10.3390/children10081346

**Published:** 2023-08-04

**Authors:** Monika Szpunar, Andrew M. Johnson, Molly Driediger, Patricia Tucker

**Affiliations:** 1 Health and Rehabilitation Sciences Program, Faculty of Health Sciences, University of Western Ontario, London, ON N6A 3K7, Canada; mszpuna@uwo.ca; 2 School of Health Studies, Faculty of Health Sciences, University of Western Ontario, London, ON N6A 3K7, Canada; ajohnson@uwo.ca; 3 School of Kinesiology, Faculty of Health Sciences, University of Western Ontario, London, ON N6A 3K7, Canada; mdriedig@uwo.ca; 4 Children’s Health Research Institute, Lawson Health Research Institute, London, ON N6C 2R5, Canada; 5 School of Occupational Therapy, Faculty of Health Sciences, University of Western Ontario, London, ON N6A 3K7, Canada

**Keywords:** outdoor risky play, early childhood educators, childcare, self-efficacy, risk tolerance

## Abstract

Children’s outdoor risky play is important for healthy development. However, Early Childhood Educators (ECEs) concern for child safety often restricts risky play affordances during childcare. To reduce this trend, an Outdoor Play Risk Re-Framing workshop was delivered to ECEs in London, Ontario, and the immediate/short-term impact of the workshop on ECEs’ knowledge, self-efficacy, and risk tolerance for engaging children in outdoor risky play was examined. Via a natural experiment, using a quasi-experimental design, ECEs in the experimental group (*n* = 119) completed an Outdoor Play Risk Re-Framing workshop, while ECEs in the comparison group (*n* = 51) continued their typical curriculum. All ECEs completed the same survey assessing their knowledge (*n* = 11 items), self-efficacy (*n* = 15 items), and risk tolerance (*n* = 27 items) at baseline and 1-week post-intervention. A maximum likelihood linear mixed effects model was conducted, while deductive content analysis was used for open-ended items. The workshop intervention resulted in significant improvements in ECEs’ self-efficacy (*p* = 0.001); however, no significant changes were observed for knowledge (i.e., awareness and practices; *p* = 0.01 and *p* = 0.49, respectively) or risk tolerance (*p* = 0.20). Qualitative data revealed similar findings across both groups, highlighting physical development as a benefit to outdoor risky play and fear of liability as a barrier. In conclusion, providing ECEs with an Outdoor Play Risk Re-Framing workshop shows promise for supporting their self-efficacy to promote this behavior but does not impact ECEs’ knowledge or risk tolerance to lead outdoor risky play.

## 1. Introduction

Physical activity engagement from birth to 5 years is important for proper growth and development [1]. Not only is physical activity associated with increased cardiovascular, musculoskeletal, and cognitive health in children [2], but it also reduces the likelihood of developing chronic conditions such as anxiety and depression in later life [1,3]. More recently, research exploring the benefits of physical activity on children’s health has revealed the importance of participating in outdoor play on various aspects of physical and mental well-being [4] and has underscored its association with increased levels of physical activity [5]. Furthermore, engaging in outdoor play has been found to support children’s emotional and behavioral regulation and aid in the development of independence [6]. In Canada, many children (~66%) are being cared for in childcare facilities [7], and research has shown that children are not engaging in sufficient physical activity in these settings [8]. Further, outdoor time during childcare hours is often limited for various reasons, some of which include inclement weather, time restraints (e.g., sharing an outdoor yard at daycare facilities with other classrooms), and/or personal values towards outdoor play among Early Childhood Educators (ECEs) [9]. For these reasons, it is necessary that the childcare setting is well-equipped to provide children with plentiful opportunities to become active and encourage various forms of movement, including outdoor play.

The outdoor environment in childcare settings has been identified as significantly influencing children’s play experiences [9,10], as children are more active outdoors versus indoors during childcare [11]. Outdoor settings possess opportunities for children to engage in challenging activities (e.g., climbing trees, monkey bars, playing in open fields) due to the presence of many naturally occurring elements that appeal to children (e.g., rockfaces, puddles, fields) [12,13]. Outdoor activities often involve inherent risks; therefore, engaging in this behavior is commonly termed *risky play* [14]. Engaging in risky play offers numerous health benefits for children, including resilience building, motor skill development, and increased confidence [13]. Further, it provides children with opportunities to test their personal limits, learn to make decisions surrounding injury and risk, and challenge themselves and each other [14]. Many types of outdoor activities can involve risky play, and these activities have previously been categorized as play at speed, play at height, play with dangerous tools, play near dangerous elements, rough and tumble play, and play where children can hide or become lost [14], many of which are possible for children to engage in during childcare hours, specifically while outdoors.

Considering the large enrolment of children under 5 years of age in Canadian childcare settings [7], a growing concern exists regarding whether children are permitted to engage in or are provided with diverse outdoor play opportunities within these settings [12,15]. In fact, numerous studies have identified that children’s opportunities for outdoor play that involve risk are low or diminishing, and this may be due to increasingly risk-averse societies [16,17] that rely on technology, such as screen-viewing (e.g., television, video games) to keep children occupied [18]. With respect to the childcare environment, heightened fears have been observed by childcare staff (i.e., ECEs, and directors) due to safety regulations and concerns for parental discernment and liability in cases if an injury were to occur [19]. As a result, children are regularly kept inside, limited to predominantly indoor activities, and directed by adults [20]. This limits children’s opportunities to reap the abovementioned benefits associated with outdoor play and risk [13].

ECE’s personal beliefs and their level of training regarding outdoor play are also important to consider [21] as their behavior toward leading and promoting various types of children’s play often reflects their own personal meaning that they place on this behavior [22]. Specifically, the importance of ECE’s opinions toward facilitating outdoor play involving risk has been previously established [23,24,25]. For example, Stephenson et al. (2003) found that when ECEs’ personal attitudes, perspectives, and opinions surrounding outdoor play were positive, and when they personally valued being outdoors, they were more likely to support children’s outdoor risky play. Despite these findings, evidence supports that this type of play has not been offered frequently enough in early childhood education settings [26,27]. For example, a recent childcare-based study conducted by Sandseter et al. (2020) in Norway identified that only 10.3% of children’s time designated for free play was spent engaged in risky play, suggesting that increased facilitation from ECEs and childcare center staff is needed [28]. Finally, it is important to note that ECEs themselves have noted their desire for increased training regarding the promotion of healthy movement behaviors in early childhood education curriculums [21], and professional learning approaches have been found to be effective at supporting improved knowledge and self-efficacy in ECEs [29]. For example, following the completion of a 5 h e-learning course that taught content regarding healthy movement behaviors during childcare, including outdoor and risky play, ECEs significantly improved their total knowledge, self-efficacy, perceived behavioral intention, and control scores [29]. This finding aligns with a recent scoping review by Mak et al. (2021) which identified multi-strategy approaches, such as those that combine professional development training opportunities with continuous follow-up (e.g., continued support after the professional development period) as promising for increasing ECEs’ ability to promote movement for children under their care [30].

Recognizing that promoting outdoor risky play can be a challenging task for ECEs to perform is important [19]; thus, educating ECEs on the significance of this activity for young children’s health is a crucial first step. In addition to increasing their knowledge of the importance of promoting children’s outdoor play involving risk, strategies to ensure ECEs have self-efficacy or confidence in their ability to deliver such tasks must also be provided. Evidence shows that having sufficient preparatory knowledge about the importance of behavior, and understanding how to facilitate it, increases confidence in one’s ability to complete a task [31]. Consequently, nurturing ECEs’ self-efficacy regarding physical activity promotion has been noted as especially important to improve children’s physical activity and play affordances in childcare [8,21,32]. Offering ECEs the opportunity to participate in professional development opportunities, such as single or multisession workshops, is an effective way to increase their knowledge and produce meaningful change in their skills and behaviors [33]. More specifically, training opportunities (e.g., professional development and learning) can help build ECE’s skills and encourage children to engage in healthy behaviors, including outdoor risky play [34].

In an attempt to reduce ECE’s fears toward supporting children’s healthy engagement in risky play while outdoors, risk re-framing has been identified as necessary to change perspectives on outdoor play [35]. Risk re-framing aims to modify the perception of risk and change behaviors, and has previously been developed and tested for the parents and teachers of elementary school children (5–7 years; [36]) with positive outcomes [37], and more recently, for mothers of children aged 6 to 12 years [35,38]. However, to our knowledge, the efficacy of a Risk Re-Framing workshop tailored specific for ECEs has not yet been explored. Therefore, in partnership with a local childcare agency, the purpose of this study was to examine the immediate/short-term impact of an Outdoor Play Risk Re-Framing workshop on ECE’s knowledge, self-efficacy, and risk tolerance for supporting children’s outdoor risky play in center-based childcare. A secondary objective was to explore ECEs’ perspectives, including specific benefits, fears, barriers, and facilitators regarding the promotion of outdoor risky play. To address these objectives, a survey capturing both quantitative data (e.g., to assess the impact of the workshop on ECE’s knowledge, self-efficacy, and risk tolerance) and qualitative data (e.g., to capture perspectives) was used. A mixed methods approach was employed to understand the holistic picture of ECEs’ perspectives on outdoor risky play, including meanings obtained from open-ended items as well as the prevalence of traits of ECEs obtained from surveys to enhance our understanding of the study’s findings [39]. It was hypothesized that ECEs who received the training would demonstrate greater knowledge, self-efficacy, and risk tolerance scores compared to those who did not complete the workshop.

## 2. Materials and Methods

### 2.1. Study Design and Recruitment

Using a quasi-experimental study design, this natural experiment recruited participants to the experimental group from YMCA childcare centers, while comparison group participants were recruited from other (non-YMCA) center-based childcare centers across London, Ontario. The study and related documents received approval from The Non-Medical Research Ethics Board at the University of Western Ontario (REB #113866) on 30 April 2019. All ECEs provided written consent prior to participation.

### 2.2. Development and Description of the Outdoor Play Risk Re-Framing Workshop

The Outdoor Play Risk Re-Framing workshop natural experiment, including supplementary content (e.g., handouts, PowerPoint presentation) and associated training was developed by an Outdoor Play Specialist from the Playing to Learn curriculum at the YMCA of Southwestern Ontario. The workshop and training were created using the best available evidence to guide the content covered. The workshop was created to inform childcare staff (i.e., ECEs) of the importance of implementing and scheduling time for children’s outdoor risky play during childcare hours. The Outdoor Play Risk Re-Framing workshop was delivered on a single day (via a PowerPoint presentation), which lasted 6 h, and was provided to ECEs at their respective centers by an Outdoor Play Specialist and Trainer lead. The workshop involved various group discussions (e.g., among participating ECEs), as well as individual assessments (e.g., quizzes/evaluations to test knowledge). The workshop included information on the Position Statement on Active Outdoor Play [4] and Sandseter’s six categories of risk [14] and provided an overview of provincial (e.g., Ontario’s) outdoor standards [40]. A variety of delivery approaches were employed to share the content with participants. Instructional videos were embedded into the workshop and were followed by group discussion and reflection concerning the learning outcomes. Tools such as various-sized balls, hard-boiled eggs, markers, flip chart paper, and whiteboards were used to facilitate hands-on learning. For example, hard-boiled eggs were used to facilitate play involving risk. Educators did not know that eggs were hard-boiled and were asked to pass them back and forth among their group members to simulate the risk of the unknown. ECEs were also taught about the importance of outdoor play for young children and strategies for the responsible facilitation of children’s activities involving risky play (e.g., how to play with loose parts) in the outdoor classrooms of childcare centers. At the end of the workshop and training session, a final evaluation (e.g., quiz) was distributed to test the knowledge obtained.

### 2.3. Participants

#### 2.3.1. Experimental Group

Experimental group ECEs were invited to participate if they were fluent in English and were employed full-time, part-time, or as support staff at the YMCA of Southwestern Ontario.

#### 2.3.2. Comparison Group

Comparison group ECEs were invited to participate if they were fluent in English, employed full-time, part-time, or as support staff at other London, Ontario-based childcare organizations that were not associated with the YMCA.

### 2.4. Data Collection

All data collection took place between July and December 2019. The experimental group participants’ knowledge, self-efficacy, and risk tolerance were assessed prior to receiving the Outdoor Play Risk Re-Framing workshop and at 1-week after participating in the training. Comparison group ECEs completed the same questionnaire package at the baseline and at 1-week follow-up after undergoing no change in their daily routines (e.g., no workshop provided). Participants were assigned a unique ID generated by the research assistant prior to completing the first survey, and baseline and post-intervention surveys were linked via participant IDs. Baseline surveys were completed via paper, and follow-up surveys were completed online using Qualtrics. Upon the completion of the study, comparison group ECEs were able to request information on the Outdoor Play Risk Re-Framing workshop and its associated training.

### 2.5. Measures

#### 2.5.1. Demographic Questionnaire

All ECEs were asked to complete a demographic questionnaire at the baseline, which collected their age, gender, ethnicity, level of education, the age group of children that they cared for (i.e., infant, toddler, preschooler), employment status (e.g., full-time, part-time) and previous exposure to outdoor play risky play and physical activity-related training.

#### 2.5.2. Knowledge (Awareness, Practices, and Attitudes) Questionnaire

ECEs’ knowledge was assessed using a questionnaire comprised of 3 sections (e.g., awareness, practices, and attitudes), which was developed for the purpose of this study. Awareness items were phrased as questions (*n* = 4; e.g., “*The best example of unstructured challenging, or outdoor risky play in childcare is*”), and reflected topics that were covered in the Outdoor Play Risk Re-Framing workshop. Awareness items were asked via a multiple-choice format (e.g., only one correct answer per question that ECEs had to identify). A higher total score (out of 4) indicated greater awareness. In addition, ECEs were asked to report on their personal practices (*n* = 3) which they engaged in during a typical childcare day (e.g., “*To what degree do you currently implement unstructured challenging, or outdoor risky play with your group of children?*”*)* for each item on a Likert scale from 1 (not at all) to 5 (completely). Finally, four open-ended questions were included at the end of the knowledge questionnaire and asked ECE’s attitudes toward benefits, fears, barriers, and facilitators regarding outdoor risky play (e.g., “*What are the barriers to engaging young children in unstructured challenging, or outdoor risky play in childcare?*”*)*. These questions were used to gather more in-depth and descriptive perspectives that could not be captured via quantitative items. The knowledge items used in the present study were informed by items included in Brussoni and colleagues’ (2018) digital tool for assessing the mother’s tolerance toward children’s risk play [38]; however, these were modified to represent instances in the childcare setting.

#### 2.5.3. Self-Efficacy Questionnaire

ECEs’ self-efficacy to engage children in outdoor risky play was assessed with items (*n* = 15) that were developed based on the content of the training and the existing literature surrounding ECEs’ attitudes toward outdoor risky play (e.g., “*At this moment, I am confident that I can support children’s engagement in unstructured challenging, or outdoor risky play despite potential judgment from parents*”). The items were created to represent two constructs: (1) task self-efficacy: or the confidence one feels to engage children in outdoor risky play, and (2) barrier self-efficacy: the confidence one feels to overcome barriers associated with engaging children in outdoor risky play. More specifically, barrier items were created to represent three separate constructs: barriers related to the childcare environment, barriers related to supervision, and barriers related to the concerns or perceptions of others. ECEs were asked to rank items on a Likert scale from 0 (not at all confident) to 100 (completely confident). Higher self-efficacy scores indicated greater confidence to engage children in outdoor risky play.

#### 2.5.4. Tolerance for Risk in Play Scale

This valid and reliable 31-item survey was originally developed to assess adults’ tolerance for outdoor risky play among children 3 to 13 years [41]. While this survey’s validity and reliability (i.e., Pearson reliability index of 0.87; [40]) have previously been demonstrated with primarily parent respondents (a small number of elementary school teachers were included in the original sample), its psychometric properties have yet to be determined with ECEs. The wording of the items was revised to reflect the childcare setting and its administration to ECE respondents (e.g., “*Would you allow the children in your care to…*”). Items that portrayed situations that were not possible in childcare, or those that neglected childcare safety standards, were removed. ECEs were asked to respond to each item with a “yes” or “no”. A space for an optional comment was provided at the end of each question on the tolerance of the risk questionnaire. A higher total score (calculated by summing the number of “yes” responses) indicated greater tolerance for risk during children’s outdoor play.

### 2.6. Data Analysis

All statistical analyses were performed in R (version 4.0.4; [42]). Descriptive statistics, including means and standard deviations, were calculated to describe participant demographics. To ensure age and sex comparability between the groups, independent sample t-tests and Pearson chi-square tests were completed. Composite scores were created as unit-weighted composites for all outcome variables (e.g., knowledge, including practices, self-efficacy, and risk tolerance). Awareness scores, under the knowledge section of the survey, were calculated as the sum of the correct answers to the 4 questions. Self-efficacy was the average of self-efficacy items (*n* = 15), while practices referred to the average of practice items (*n* = 3), and risk tolerance was the average of risk tolerance items (*n* = 26).

To explore the effect of the interventions and their immediate/short-term impact on knowledge, self-efficacy, and risk tolerance, a maximum likelihood linear mixed effects model was utilized, with a group (experimental versus comparison) and time (pre- versus post-) entered as fixed effects. Linear mixed effects analyses were conducted using the lme4 [43] and car [44] packages. All possible comparisons amongst the time periods were assessed using the emmeans package [45]. A Bonferroni correction was used to adjust for multiple comparison bias. Three analyses were performed (knowledge, self-efficacy, and risk tolerance); therefore, alpha was adjusted to 0.05/4 = 0.0125. Open-ended questions were analyzed via deductive content analysis [46] using QSR Nvivo (version 12). Deductive content analysis was conducted by two trained research assistants to identify common responses (e.g., themes) and ensure confirmability [47]. The responses were deductively analyzed by a question asked, which was in line with the online questionnaire.

## 3. Results

A total of 170 ECEs (*n* = 119 experimental; *n* = 51 comparison) were enrolled in this study. The majority of the experimental group ECEs were Caucasian (73.1%), employed full-time (94.1%), and had a college degree (71.4%). The majority of comparison group ECEs were also Caucasian (80.4%), employed full-time (96.1%), and had a college degree (80.4%). More ECEs in the experimental group reported having previous physical activity and outdoor play-related training when compared to the comparison group. See Table 1 for full participant demographics by each group.

### 3.1. ECEs’ Self-Efficacy, Knowledge, and Risk Tolerance

The means and standard deviations for knowledge (separated by awareness and practices), self-efficacy, and risk tolerance, separated by time and group, are presented in Table 2. There was a significant change in self-efficacy F (1, 117.14) = 11.11, *p* = 0.001, with ECEs in the experimental group reporting a significant increase across time. For knowledge, no significant change between the pre- and post-test in terms of awareness scores was observed F (1, 123.03) = 6.35, *p* = 0.013 after controlling for a multiple comparison bias. Similarly, there was no significant change between the pre- and post-test in the practices category F (1, 124.25) = 0.49, *p* = 0.4868. Finally, group differences were observed for risk tolerance scores; however, when time was added as a factor, significance was lost. As such, there was no significant improvement in risk tolerance because of the Outdoor Training Workshop, F (1, 112.97) = 1.65, *p* = 0.20.

### 3.2. ECEs’ Attitudes of Benefits, Fears, Barriers, and Facilitators regarding Outdoor Risky Play

When asked about benefits, fears, barriers, and facilitators regarding encouraging young children’s participation in outdoor risky play, ECEs from both groups had very similar perspectives. ECEs expressed that they noticed many benefits, citing both physical (e.g., improved hand-eye coordination) and psychological (e.g., gaining self-confidence) benefits in children from engaging in outdoor play involving risks. Distinct perspectives from ECEs regarding their fears and barriers during promoting outdoor risky play identified via open-ended responses included: concern for children becoming injured or hurt under their supervision (and fear of liability), children taking risky play too far, lack of space at their respective childcare centers, and disapproval from parents, and other ECEs. ECEs reported that having proper materials (e.g., loose parts), being near the children during their participation in outdoor risky play and having sufficient outdoor space at their childcare centers were effective facilitators for the aforementioned fears/barriers. ECEs also noted their role and reported being aware of their actions (e.g., role modeling outdoor risky play) as examples of why they felt equipped to support this behavior during childcare hours. See Table 3 for ECEs’ perspectives regarding perceived benefits and fears, barriers, and facilitators to outdoor risky play as grouped by the open-ended questions.

## 4. Discussion

The primary objective of this study was to examine the immediate/short-term impact of an Outdoor Risk Re-Framing workshop on the knowledge, self-efficacy, and risk tolerance of ECEs working in center-based childcare. While the results of this study reveal that the workshop was successful at increasing the experimental group ECE’s self-efficacy, no statistically significant results were noted for knowledge or risk tolerance. Several findings from this study are considered below.

The research shows that the level of engagement regarding ECEs’ facilitation of physical activity for children is dependent on many factors, including their level of education, training, experience, and personal beliefs [21,32,46]. ECEs in Canada have previously acknowledged their limited training regarding physical activity and outdoor risky play [21]; consequently, it was not surprising that increases in self-efficacy were observed by ECEs who attended the Outdoor Play Risk Re-Framing workshop. This may be due to the education surrounding outdoor risky play that was delivered to ECEs and the increase in confidence that was obtained as part of the workshop, and because of the high self-efficacy scores that were observed at the baseline. Although we cannot be certain that the increase in self-efficacy was due to the workshop, the observed positive impact on self-efficacy at post-training aligns with Copeland and colleagues’ recommendation that increased training and support for ECEs is crucial for physical activity-related interventions and can help to increase ECEs’ self-efficacy [22]. Currently, there is a strong curricular focus placed on non-movement activities during a typical childcare day, such as reading or circle time, that serve to prepare children for the transition to school [48]; thus, ECEs may not identify physical activity or risk-taking behaviors as essential programming components [22,49,50]. As such, providing ECEs with outdoor risky play training outside of traditional early childhood education milestones (e.g., degrees) may serve as a possible solution to inform ECEs of the importance of this behavior and to ensure they are well-equipped to support these behaviors and feel competent to do so.

In the present study, nearly half of all participating ECEs reported having previous physical activity and previous outdoor play-related training. This is interesting, as a Canadian study found that ~32% of early childhood education students (*n* = 1292) reported receiving previous physical activity-related training during their college and university education [21]; however, when breaking down the components of these curriculums, not all of them included content regarding outdoor risky play. Therefore, it was unexpected that more ECEs in the present study reported having previous outdoor training compared to physical activity training. This finding could be a consequence of the large amount of center-based childcare physical activity-related research projects being conducted in the London, Ontario childcare community (e.g., SPACE, PLAY, LEAPP) [51,52,53], which have training embedded as a part of intervention procedures, as these research studies were frequently noted as the previous training received when ECEs were asked to report who provided the training.

While knowledge scores in both the awareness and practices categories may not have been significantly impacted by the Outdoor Play Risk Re-Framing workshop, the experimental group ECEs’ awareness scores were already very high at the baseline and still showed a slight increase at post-intervention. This suggests that the workshop may have increased knowledge; however, further research with a larger sample of ECEs is needed to support this claim. Similarly, although risk tolerance scores did not change between the pre- and post-test, findings from the present study revealed that ECEs in both experimental and comparison groups, regardless of training, believed that outdoor risky play was important and beneficial for the children in their care. These findings are consistent with a study by McFarland et al. (2018), whose research identified that ECEs in Australia and the United States believed outdoor risky play to be critical for children’s overall development and believed themselves to be key individuals responsible for providing appropriate risky play opportunities [54]. Conversely, the ECEs enrolled in McFarland and colleagues’ (2018) study identified no barriers to providing outdoor risky play opportunities, which contrasts with our findings. ECEs in the present study identified fear of parental complaints (e.g., children coming home with dirty clothes), general safety concerns, and worry regarding the attitudes of other ECE colleagues as barriers to implementing outdoor risky play during care hours. These fears and/or barriers may serve as the culprit for the lack of change in risk tolerance scores. As such, future training on how to overcome barriers is needed for future workshops and training in this area. Promoting education opportunities for ECEs surrounding outdoor play activities that involve risk and/or professional development (e.g., webinars) and teaching how to facilitate these behaviors may be an effective strategy to foster increased tolerance for risk among ECEs.

ECEs in the present study reported their own personal investment placed in keeping children safe as an important factor when considering and encouraging outdoor risky play. This type of personal investment has been identified in studies involving risky play perceptions with parents [55] but has not been commonly reported in studies with ECEs [54]. ECEs in the present study noted their unique childcare environments as both barriers and facilitators to engaging children in outdoor risky play. Similar findings were noted by Little et al. (2012), who found that Australian and Norwegian ECEs viewed the quality of their outdoor environments as barriers to proper implementation. These findings are important as they serve as a call to action for how we can better equip ECEs and support them in their important role of facilitating outdoor risky play by providing materials (e.g., loose parts) and educating ECEs on how to make the most of their respective childcare center’s outdoor space, which can ultimately aid in their daily outdoor risky play programming [56]. Future studies should move beyond survey-only measures to assess changes in ECEs’ self-efficacy, knowledge, and risk tolerance regarding outdoor risky play. For example, interviews or focus groups with ECEs could inform the content that could be included in future workshops or training sessions and provide important insights from the target audience (i.e., ECEs) regarding the training format, delivery, and time commitment. In addition, qualitative research could solicit more in-depth descriptions of ECEs’ perceived barriers to implementing outdoor risky play during childcare. This is important as understanding perceived barriers could help inform solutions and provide training content regarding the promotion of outdoor risky play. Finally, following the identification of such barriers and solutions, an intervention based on these findings could be created to assess identified strategies (e.g., solutions) for encouraging ECEs to promote outdoor risky play in childcare.

The strengths of this study include the quasi-experimental study design employed, the use of open-ended items to further understand ECEs perspectives, and the use of a valid, reliable tool for assessing the risk tolerance of ECEs [41]; however, this study is not without its limitations. Firstly, all data were self-reported; thus, a social desirability bias may have been present. In addition, the definition of risky play was not defined for comparison group participants (i.e., no workshop received); thus, it was possible that experimental and comparison group ECEs had varied understandings of outdoor risky play. Further, measurement periods were only 1 week apart, and many participants from the experimental group did not complete the second survey; thus, the reported findings of the intervention represent a smaller sample and effect size than was intended. It is possible that participants had a strong memory effect considering the measurements that were taken only 1-week after the workshop. Of note, the workshop was only 1-day (i.e., 6 h); thus, it may have been too brief to lead to significant changes in knowledge, self-efficacy, and risk tolerance. Moreover, the difference in the number of participants in the experimental and control groups also acts as a limitation, as does the homogeneity of demographics between these groups. For example, ECEs in both groups identified as mostly Caucasian—80% and 73% in comparison with the experimental group ECEs, respectively—and nearly all ECEs had full-time employment and college degrees; therefore, our ability to examine the impact of demographic factors on the study results was limited. Given that no valid, reliable tool exists which can measure ECEs’ physical activity and outdoor risky play-related self-efficacy, the measurement method used in the present study was created by the researchers and is, thus, not officially validated. To facilitate a parsimonious description of the effects within this study (specifically, changes over time), we aggregated item information to create composite scores for our knowledge, self-efficacy, and risk tolerance scores. The aggregation of item scores within such composites may obscure individual differences in specific facets of the construct being studied. A More in-depth study of these facets was, however, beyond the scope of the present study. In addition, nearly half of the participants reported receiving previous physical activity and outdoor play-related training, and this was not accounted for in our analysis, which may have influenced the findings. Of note, ECEs from the experimental group received prior exposure to outdoor risky play settings (as a part of the Playing to Learn curriculum at the YMCA) and likely had previous training in relation to the use of loose parts and outdoor playgrounds, which may have led to higher baseline scores. Therefore, we cannot conclude that the increased self-efficacy observed in the present study was due to the workshop alone, and the follow-up measurements were not long; therefore, the effect was not sustained. Finally, all participants were purposefully sampled from a group of ECEs from London, Ontario, thus limiting generalizability. Additional research with a larger sample is necessary to confirm these findings and demonstrate an association between Outdoor Risk Re-Framing workshops and ECE’s knowledge, self-efficacy, and risk tolerance.

## 5. Conclusions

The results of this study suggest that attending an Outdoor Play Risk Re-Framing workshop holds promise as a strategy for increasing ECEs’ self-efficacy regarding outdoor risky play; however, this needs further, more rigorous exploration with longer periods of measurement. Future research should seek to explore how early childhood education settings can adopt outdoor play spaces that support and accommodate diverse opportunities for children to engage in risky play. It is important to acknowledge that children’s levels of comfort with risky play may vary; therefore, more research is needed to identify what factors influence these variations (e.g., children’s age, number of siblings, exposure to free play in nature).

## Figures and Tables

**Table 1:** Descriptive characteristics of Early Childhood Educators by group.

Variable	Comparison (*n* = 51)	Experimental (*n* = 119)	*p*-Value
*Age (years), M (SD)*	40.1 (13.4)	31.0 (8.9)	0.00
*Sex (female, male), n*	50.1	114.5	0.47
	N (%)	N (%)	
*Years of experience as an ECE*			0.000
Less than 5 years	9 (17.6)	63 (52.9)	
5 to 9 years	10 (19.6)	28 (23.5)	
10 to 14 years	6 (11.8)	9 (7.6)	
15 to 20 years	7 (13.7)	10 (8.4)	
20+ years	19 (37.3)	9 (7.6)	
*Ethnicity*			0.43
Caucasian	41 (80.4)	87 (73.1)	
African Canadian	1 (2.0)	1 (0.8)	
Native/Aboriginal	0 (0)	1 (0.8)	
Arab	1 (2.0)	2 (1.7)	
Asian	4 (7.8)	16 (13.4)	
Latin American	1 (2.0)	4 (3.4)	
Other	0 (0)	6 (5.0)	
Prefer not to answer	3 (5.9)	2 (0.17)	
*Employment status*			0.76
Full time	49 (96.1)	112 (94.1)	
Part time	2 (3.9)	6 (5.0)	
Occupational	0 (0)	1 (0.8)	
*Age group of children*			0.51
Infants	12 (23.5)	24 (20.2)	
Toddlers	16 (31.4)	27 (22.7)	
Preschoolers	14 (27.5)	39 (32.8)	
Multiple age groups	9 (17.6)	29 (24.4)	
*Highest level of education*			0.54
High school	1 (2.0)	5 (4.2)	
College	41 (80.4)	85 (71.4)	
University	9 (17.6)	27 (22.7)	
Graduate school	0 (0)	2 (1.7)	
*Previous physical activity-related training?*			0.67
Yes	20 (39.2)	50 (42.7)	
No	31 (60.8)	67 (57.3)	
*Previous outdoor play training?*			0.12
Yes	19 (37.3)	60 (50.4)	
No	32 (62.7)	59 (49.6)	

Note. Data are reported for participants who completed the demographic survey. All values shown may not add up to 100% or *n* = 119 (experimental) or *n* = 51 (control) due to incomplete data. Groups were compared using independent *t*-tests for continuous variables and Pearson-chi tests for categorical variables.

**Table 2:** Means (standard deviations) of knowledge, self-efficacy, and risky tolerance scores for participating ECEs.

Time	Knowledge				Self-Efficacy		Risk Tolerance	
	Awareness		Practices					
	Comp	Exp	Comp	Exp	Comp	Exp	Comp	Exp
**Pre**	3.51 (0.67)	3.55 (0.63)	3.44 (0.63)	4.05 (0.53)	63.80 (16.13)	69.41 (14.86)	0.53 (0.16)	0.65 (0.17)
**Post**	3.55 (0.75)	3.89 (0.36)	3.64 (0.54)	4.30 (0.52)	61.92 (17.04)	75.10 (13.17)	0.54 (0.16)	0.69 (0.15)

Note. Exp = Experimental group. Comp = Comparison group. Reported differences for each time period represent differences between groups.

**Table 3:** ECEs’ perspectives of benefits, fears, barriers, and facilitators regarding outdoor risky play.

Question	Sentiments by Group	
	Experimental	Comparison
*What do you feel are the benefits to supporting outdoor risky play?*	“I can feel the benefits on the children in my classroom in so many ways. I see them solving problems on their own, overcoming fears and gaining self-confidence.” “Children learning new skills, knowing their limits, overcoming fears, and gaining independence—these are the benefits of teaching risky play.” “They build their self-esteem.” “When you see the big smile that a child makes at the end of accomplishing a tricky task, it makes you happy too.”	“I think it is important to allow children to figure out their comfort zones.” “It helps them learn to problem solve.” “Children will learn what is dangerous.” “I love seeing the children push each other and end up working together.” “Preparing children for later life.” “Improved hand eye co-ordination.”
*What concerns you about encouraging your children to engage in outdoor risky play? (e.g., fears)*	“I am worried about complaints from parents.” “Children getting injured, or parents being upset when seeing their child engaging in risky play.” “Negative reactions from parents.” “Not being close enough in the case that something bad were to happen.”	“I am concerned about choking hazards, broken bones, and head injuries.” “I am concerned that not all parents or staff will be supportive and am also worried about my own nervousness.” “Fear that some children will take it too far.” “Having to explain an injury to a parent or our director.”
*What do you feel are the barriers to supporting outdoor risky play?*	“Childcare directors’ opinions.” “Different abilities of the children I care for.” “Sometimes the loose parts from our playgrounds are removed.” “Rules at my daycare.” “Some parents over-protect their children.” “Lack of support from other educators.” “Unsafe areas and limits on what we can allow.”	“Lack of materials at my childcare centre.” “Coworkers who disagree.” “Parents who have said they do not approve of risky play.” “Parents not wanting their children to go home dirty.” “No equipment.” “There is no policy about risky play in our daycare.” “Sometimes children don’t want to try scary tasks.”
*In what way are you prepared to support children’s engagement in outdoor risky play?*	“Don’t set boundaries for the kids, if they feel like it, they will explore more.” “Provide them help if they need it.” “Access to materials like loose parts.” “Having tools such as safe scissors that I feel comfortable letting the children learn to use.”	“I feel prepared when I am close and observing what activity they [the children] may be trying.” “Role modelling.” “It helps me when the staff work together and share similar perspectives on risky play.”

## Data Availability

Data can be made available upon request.
